# Effect of macadamia oil cake on blood lipid characteristics and intestinal microbiota in hyperlipidemic rat

**DOI:** 10.1002/fsn3.3490

**Published:** 2023-06-14

**Authors:** Yao Liu, Tengfei Xie, Shijun Wu, Guang Yang, Jinyun Zhang, Jie Song, Guifang Yang

**Affiliations:** ^1^ Guangdong Eco‐engineering Polytechnic Guangzhou China

**Keywords:** blood lipid, hyperlipidemia, macadamia oil cake

## Abstract

Macadamia oil cake (MOC) is a type of macadamia nut by‐product, that is extremely rich in amino acids and has beneficial health effects. It lowers blood lipid levels and regulates the intestinal microbiota. MOC effectively attenuated total cholesterol (TC), triglyceride (TG), low‐density lipoprotein cholesterol (LDL‐C), and high‐density lipoprotein cholesterol (HDL‐C) levels in model rats. Depending on the morphology of the colon, MOC can effectively attenuate damage to the tissue structure. The 16S rDNA gene of the rat intestinal microbiota was sequenced using Illumina PE250 high‐throughput sequencing technology, and the changes in the intestinal microbiota in each group are discussed. Supplementing MOC at different doses significantly increased the microbiota of *Dorea*, *Erysipelotrichaceae*, *Stercoris*, etc. in the intestinal tracts of rats fed a high‐fat diet. Therefore, MOC can be included in lipid healthy dietary patterns to lower lipid characteristics and restructure the intestinal microbiota. Future clinical trials are required to determine the therapeutic effects and mechanisms of hypolipidemia.

## Graphical abstract

Macadamia oil cake (MOC) can effectively attenuate the total cholesterol (TC), triglyceride (TG), low‐density lipoprotein cholesterol (LDL‐C), and high‐density lipoprotein cholesterol (HDL‐C) of the model rats and show that MOC can be included in a lipid‐healthy dietary pattern to lower lipid characteristics and restructure intestinal flora.

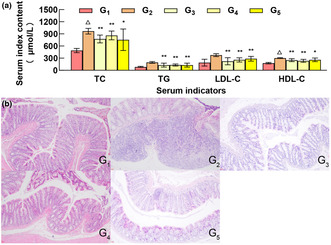

## 1 INTRODUCTION

The incidence of metabolic diseases such as hyperlipidemia, obesity, diabetes, and heart disease increases rapidly with lifestyle changes (Jia et al., 2021). Hyperlipidemia is a chronic disease characterized by high levels of total cholesterol, low‐density cholesterol, and triglycerides, and low levels of high‐density cholesterol (Deng et al., 2019). Accumulating evidence suggests that hyperlipidemia is affected by both genetic and environmental factors. Many studies have shown that the functional properties of cereals and traditional Chinese medicine, such as polysaccharides, peptides, polyphenols, and flavonoids, can improve lipid distribution in the body and alleviate intestinal disorders.

Emerging evidence has shown that there is a potential interaction between gut microbiota and hyperlipidemia disease (Chen et al., 2013). Disturbances in the intestinal microbiota can also lead to disturbances in liver–intestinal circulation and further aggravate dyslipidemia in rats and humans (Bäckhed et al., 2007; Baigent et al., 2005). Diabetic db/db mice with lipid metabolism disorders have lower levels of butyrate‐producing bacteria and higher levels of opportunistic pathogens than healthy mice (Qin et al., 2012). Similarly, humans with hyperlipidemia express low levels of fecal acetate, butyrate, and propionate, which are related to SCFA‐producing bacteria such as those from the families Lachnospiraceae and Ruminococcaceae, among others (Gargari et al., 2018). The feces of hyperlipidemic rats contain significantly more lipopolysaccharide‐producing bacteria (e.g., *Bilophila* and *Sutterella*) and microinjury bacteria (*Bilophila* and *Akkermansia muciniphila*) (Song et al., 2017). Other studies have indicated that the intestinal microbial structure of hyperlipidemic rats changes, thereby reducing the excretion of bile acids in the cecum (Han et al., 2019; Pan et al., 2018). Intestinal microbial metabolites such as short‐chain fatty acids (SCFAs), lipopolysaccharides (LPS), and bile acids (BAs) play important roles in the regulation of hyperlipidemia (Jia et al., 2021). Therefore, modulation of the abundance and composition of the gut microbiota has become a beneficial strategy for regulating lipid metabolism and preventing the development of hyperlipidemia.

Protein comprises an important class of macromolecular compounds in organisms and is centrally involved in life activities (Liu et al., 2020). It is rich in components that have important physiological functions. Protein represents an important component of tissues and cells that can be used to renew and repair tissues and cells, participate in the regulation of material metabolism and physiological functions, and plays a role in oxidative energy supply (Fan et al., 2020). Protein has high nutritional value, plays key roles in the human body, and has broad market prospects. Protein powder is composed of purified soybean protein, casein, or whey protein, or a combination of these. It is made of soybean protein, whey protein, whey, and lecithin. As one of the main components, whey protein contains high levels of methionine, and it can provide nine types of amino acids necessary for the maintenance of the human body. Studies have shown that protein powder has a clear blood lipid‐lowering function and regulates the composition of intestinal microbiota (Zhu et al., 2021).

Macadamia nuts are a rich source of monounsaturated fatty acids and contain a high percentage of palmitoleic acid [16:1(n‐7)]. We have found that the percentage of palmitoleic acid in macadamia nuts from different regions of Guangdong is between 9.9% and 14.2% (unpublished data). Studies have shown that a macadamia nut‐rich diet can decrease total cholesterol and LDL cholesterol and modulate the risk factors for coronary artery disease (Griel et al., 2008). After extraction of the macadamia oil, the residues are referred to as macadamia oil cake (MOC). Previous studies have demonstrated that MOC is rich in crude protein, crude fiber, estimated neutral detergent fiber, estimated acid detergent fiber, and minerals. MOC can affect the growth performance and carcass characteristics of lambs (Acheampong et al., 2017). However, there have been no comparative studies on the effects of different doses of MOC on blood lipids and intestinal microbiota. The main purpose of this study was to investigate the effects of different MOC doses on blood lipid levels, colon tissue morphology, and intestinal microbial composition in rats.

## 2 MATERIALS AND METHODS

### 2.1 MOC sample

Macadamia nuts were obtained from Yangjiang (Guangdong Province, China). After oil extraction using supercritical carbon dioxide, the MOC was used in the experimental procedures.

### 2.2 Animals and experimental design

SPF‐grade SD rats (male, average weight 180–220 g) were purchased from Southern Medical University (SCXK (Yue) 2016–0041, Guangzhou, China). The rats were maintained in a special feeding box containing sufficient fodder and potable water. The feeding environmental conditions were as follows: temperature 18–24°C, humidity 40%–70%, air change ≥8 times/hour, dark/light cycle (12 h each). After 1 week of acclimation, 10 rats were housed in two cages supplied with normal fodder as the normal control group. Forty rats were housed in eight cages (five rats per cage) and fed a high‐fat diet (model group). Normal fodder and high‐fat diet were obtained from Jiangsu Synergetic Pharmaceutical Biological Engineering Co., Ltd. After 4 weeks, the rats on the high‐fat diet were randomly assigned to four groups: (1) the model group was orally administered distilled water once daily, (2) the model group+ MOC low‐dosage group was orally treated with 1 g/kg/BW once daily, (3) the model group+ MOC medium‐dosage group was orally treated with 2 g/kg/BW once daily, and (4) the model group+ MOC high‐dosage group was orally treated with 4 g/kg/BW once daily. All animal care procedures were approved by and performed in accordance with the guidelines of the Institutional Animal Care and Use Committee of Hainan University, China.

### 2.3 Sample collection

After treatment with MOC for 4 weeks, all rats were fasted overnight and anesthetized. All the rats were sacrificed by CO_2_ asphyxiation. Blood samples were collected in sterilized tubes and centrifuged at 3500 × g for 15 min. Separated serum samples were collected. The colons from each rat were dissected and collected. Fecal samples were collected from the colon and immediately snap‐frozen. All of the samples were stored at −80°C for subsequent analyses.

### 2.4 Serum indicator analysis

The contents of TC, TG, LDL‐C, and HDL‐C in serum were determined using kits purchased from the Nanjing Jiancheng Bioengineering Institute according to the manufacturer's instructions.

### 2.5 Histological analysis

The colon samples were dehydrated for 12 h, embedded in a YD‐6D biological tissue embedding machine for 1.5 h, sectioned, spread, and baked. The sections were placed in xylene for 10 min for dewaxing followed by 100% ethanol, 95% ethanol, 80% ethanol, and purified water for 3 min each for hydration and then stained with hematoxylin dye for 30 s, differentiated with 1% hydrochloric acid alcohol for a few seconds, washed with running water for 3 min, returned blue by bluish liquid for a few seconds, washed with running water, dyed with eosin for 1 min, washed with running water, and dehydrated (by placing the section in 80% ethanol, 95% ethanol, and 100% ethanol for 15 s each). Using ultra‐clean adhesive film, the samples were observed using a white‐light microscope.

### 2.6 Sequencing and analysis

DNA was extracted from fecal samples, and the conserved region of rDNA was amplified using specific primers with barcodes. The PCR amplification was carried out according to the designated sequencing area, and the PCR products were quantified with a QuantiFluor™ fluorometer. The purified amplification products were mixed in equal amounts, the sequencing connector was connected, the sequencing library was constructed, and an Illumina PE250 device was used for sequencing. After filtering the low‐quality reads, the reads were assembled, the dual‐end reads spliced into tags, and the tags filtered to obtain clean tags. They were then clustered, the chimeric tags detected in the process of cluster comparison were removed, and the effective tags of the data were obtained. After obtaining the OTU, OTU abundance statistics based on the effective tags were calculated, and principal component analysis was conducted on the relative abundance of various groups.

### 2.7 Statistical analysis

The data were analyzed by analysis of variance (ANOVA) and the least significant difference using SPSS 20.0 software. For all analyses, p‐values <.05 were considered statistically significant, and *p* < .01 was considered extremely significant.

## 3 RESULTS AND DISCUSSION

### 3.1 Changes in blood lipids

**FIGURE 1:**
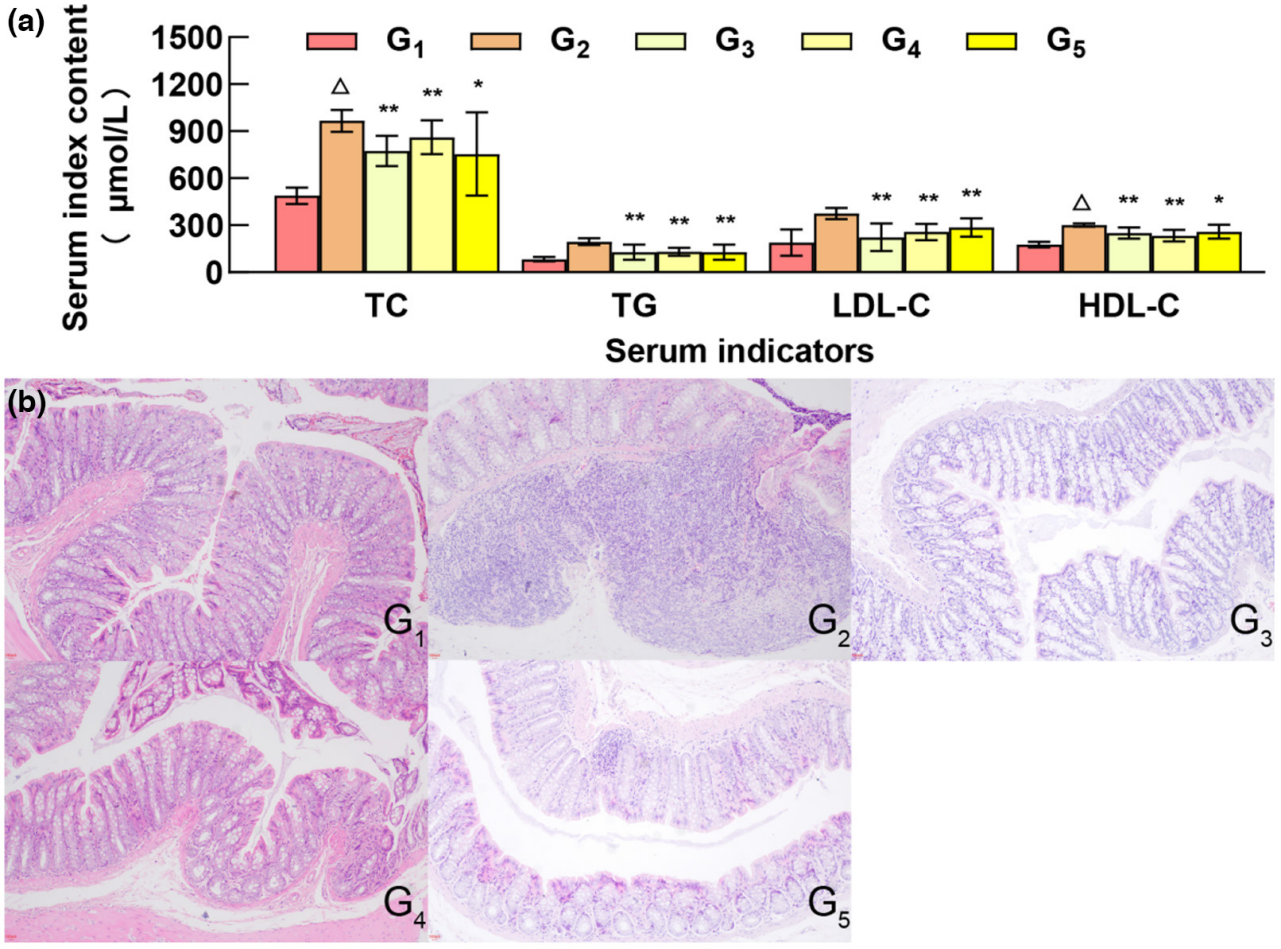
Effects of different doses of MOC on blood lipid and colon morphology in rats. G_1_, G_2_, G_3_, G_4_, and G_5_ represent the control group, model group, MOC low‐dose group, MOC medium‐dose group, and MOC high‐dose group, respectively. Compared with model group, **p < .01, *p < .05; Compared with blank group, △*p* < .01.

Proteins are the material basis of life and play important roles in various biological and physiological processes. They are necessary to maintain and repair the body and are required for cell proliferation. However, increased protein intake is not necessarily better, as excessive protein intake increases the burden on the human liver and kidneys (Song et al., 2020) and can even cause cancer (Marcello et al., 2012). As an indicator of organ function, blood lipid levels directly reflect the health status of the body (Borrego et al., 2021; Sun et al., 2015). TC, TG, LDL‐C, and HLD‐C are important indicators of blood lipid levels (Berta et al., 2021). Protein intake affects the composition and structure of the intestinal microbiota, as well as the type and level of its metabolites, thereby affecting lipid metabolism and blood lipid levels in the host (Xu et al., 2022). Compared to the control group, the TC, TG, LDL‐C, and HDL‐C levels of the model group were significantly different (*p* < .01), indicating that the hyperlipidemia model was successful. Compared to the model group, except for TC and HDL‐C in the high‐dose MOC group and HDL‐C in the model group (*p* < .05), there were significant differences between the other experimental groups and the model group (*p* < .01) (Figure 1a).

### 3.2 Colon histopathological changes

In the healthy control group (G_1_) group, the intestinal tissue structure was normal, the mucosal layer structure was complete and arranged in an orderly manner, and no necrosis or shedding of epithelial cells was observed. Inflammatory cell infiltration was not observed in the tissues, and edema was not evident in the submucosa. In the hyperlipidemia model group (G_2_) group, the intestinal tissue structure was highly abnormal, part of the crypt structure of the mucosal layer has been lost, and focal infiltration of inflammatory cells and swelling of lymph nodes were observed. In the low‐dose MOC (G_3_) group, the intestinal tissue structure was slightly abnormal, minor edema was observed in the submucosa, the tissue gap was slightly enlarged, and connective tissue hyperplasia was observed. Inflammatory cell infiltration was not observed in these tissues. In the medium‐dose MOC (G_4_) group, the intestinal tissue structure was slightly abnormal. In the visual field, the submucosa exhibited slight edema, the tissue gap was slightly enlarged, the connective tissue was proliferative, and there was no obvious inflammatory cell infiltration in the tissue. In the high‐dose MOC (G_5_) group, the intestinal tissue structure was moderately abnormal, edema was observed in the submucosa, the tissue gap was slightly enlarged, connective tissue hyperplasia and focal infiltration of inflammatory cells were observed, and the inflammatory cells were mainly lymphocytes (Figure 1b).

The results showed that the tissue structure of the normal group was essentially normal; In the model G_2_ group, the injury was the most severe, and the pathological changes were obvious. The colon damage in the high‐dose MOC group was more pronounced than that in the low‐dose group and the middle‐dose MOC group.

### 3.3 Operation classification unit and PCR analysis

**FIGURE 2:**
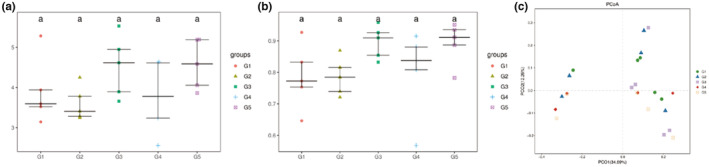
Comparison of Shannon and Simpson indexes of rat intestinal microbiota and principal coordinate analysis of their diversity. Analysis of Shannon (a) and Simpson (b) indexes and PCA (c) of intestinal microbiota in rats with different doses of MOC.

The sequencing data from the MOC group samples were clustered to produce 1115 OTUs, with an average of 465 OTUs (325–652) per sample. The α‐diversity analysis showed that compared with the healthy control group (G_1_) and the hyperlipidemia model group (G_2_), the addition of protein seeds (G_3_, G_4_, and G_5_) to the diet increased the diversity level of intestinal microbiota in hyperlipidemia mice to a certain extent, but the effect was not significant (Figure 2a,b). The PCoA‐based β‐diversity analysis results show that the microbial community structure of mice supplemented with protein seeds was more inclined to healthy mice, while the samples of mice in the hyperlipidemia model were more dispersed in the PcoA cluster (Figure 2c).

### 3.4 Analysis of intestinal microbiota composition

**FIGURE 3:**
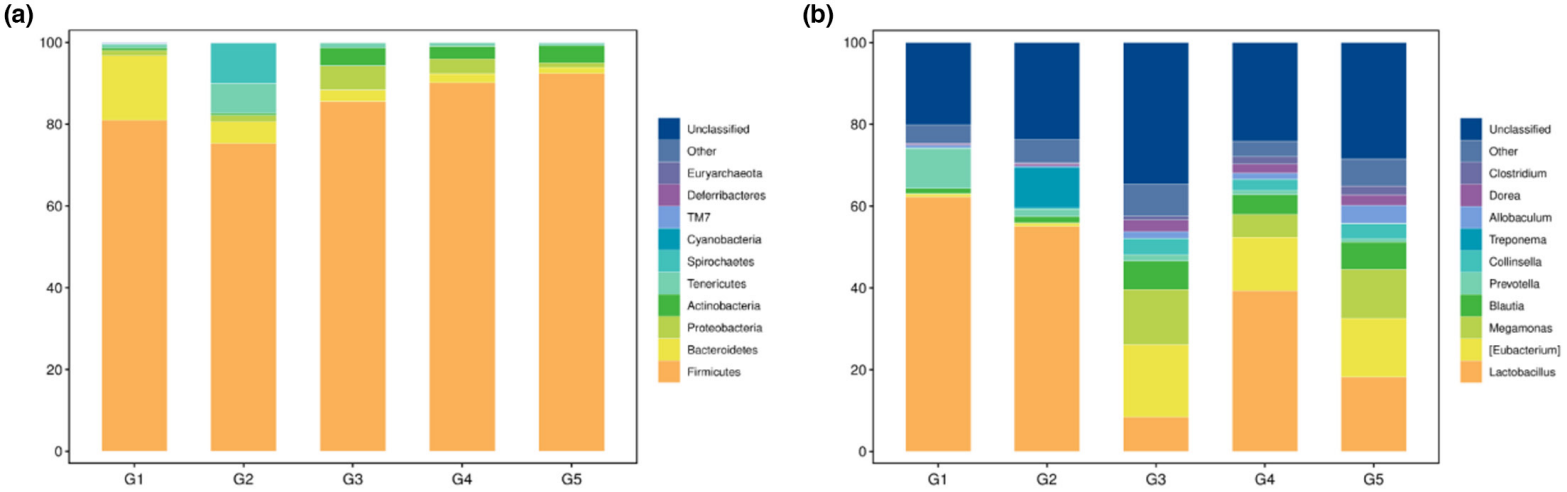
Histogram of relative abundance of species. (a) and (b) represent the relative abundance of species at the phylum and genus levels of MOC samples at different doses.

The species composition analysis results of samples with different MOC doses showed that at the phylum classification level, the relative abundance of Firmicutes and Bacteroides in the model group was lower than that in the healthy group, whereas the relative abundance of Spirochaetes and Tenericutes increased (Figure 3a). After supplementation with protein seeds, the relative abundance of Firmicutes recovered, and the relative abundance of Actinobacteria increased. At the genus level, the abundance of Treponema was significantly higher in the model group than in the healthy group. The relative abundance of Treponema was reduced after supplementation with protein seeds, while Blautia, Megamonas, and some unclassified genera increased (Figure 3b).

### 3.5 Analysis of different intestinal microbiota in rats

**FIGURE 4:**
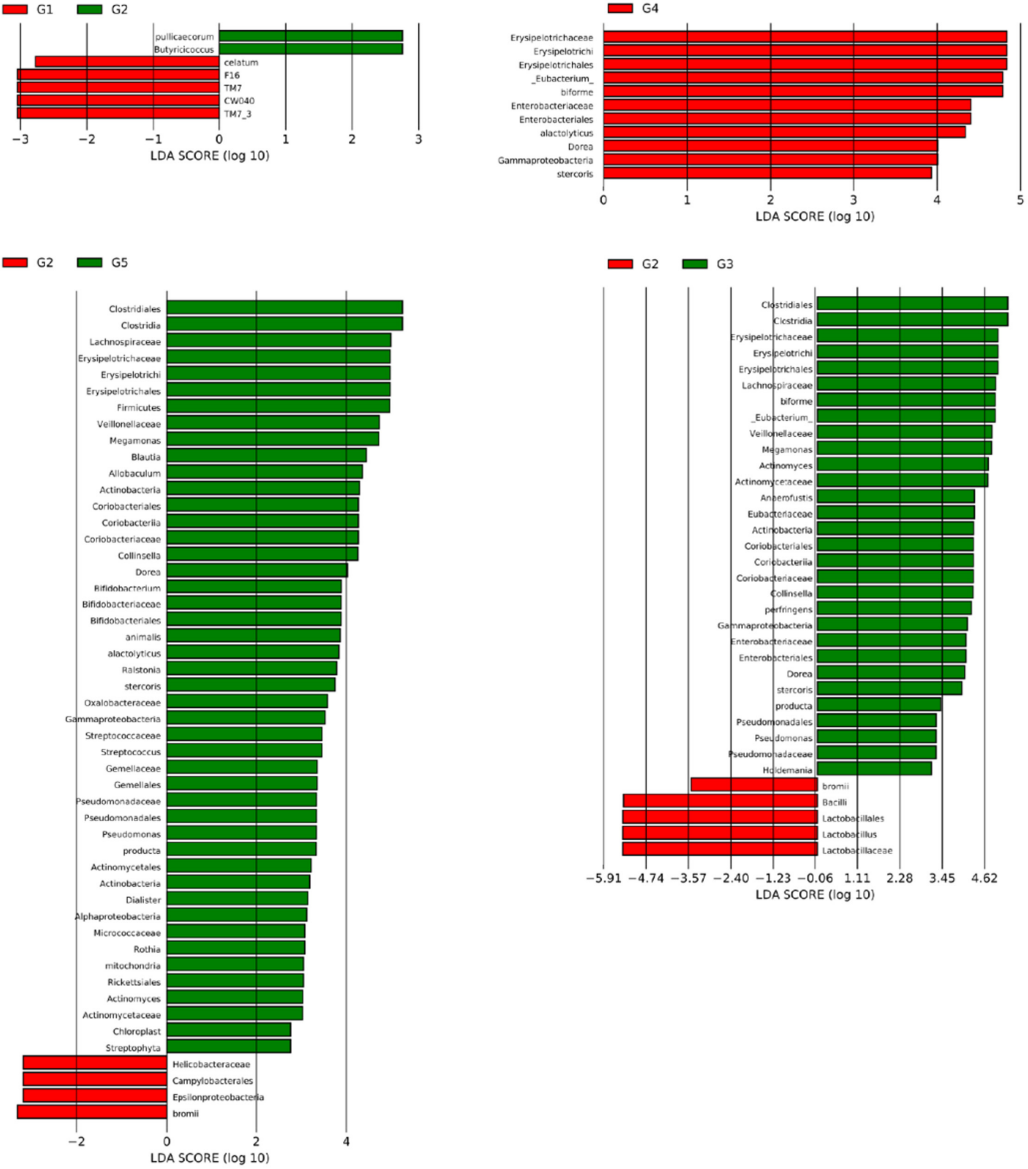
Differential microbiota analysis of different groups.

Using the LEfSe software package to analyze the different bacterial populations among groups, the results of MOC samples at different doses showed that compared with the healthy group (G_1_), *Pullicaecorum* and *Butyricicoccus* in the model group (G_2_) were significantly increased, whereas *celatum*, F16, TM7, and CW040 were significantly reduced (Figure 4). However, supplementation with MOC at different doses significantly increased a variety of bacterial populations in the intestines of high‐fat mice, including *Dorea*, *Erysipelotrichaceae*, and *Stercoris* (Figure 4) (Liang et al., 2019). A previous study has shown that protein intake can significantly affect the mTOR signaling pathway in the small intestine of rats, thereby changing the microbiota in the small intestine (Kar et al., 2017). The mTOR signaling pathway is closely related to genes involved in fat synthesis (Cao et al., 2016). High‐throughput sequencing has shown that protein intake is conducive to regulation of the intestinal microbiota of piglets and increases the proportion of beneficial bacteria (Li et al., 2021). In addition, high doses of MOC can reduce the abundance of Helicobacteraceae, Ruminococcus bromii, and other bacteria in the high‐fat rat microbiota.

In conclusion, we systematically studied the effects of different MOC doses on blood lipid levels, colon morphology, and intestinal microbiota in rats. The results show that the blood lipid levels of the mice decreased significantly after ingestion of MOC. The results of colon H&E staining showed that moderate and low doses of walnut protein effectively reduced colon injury caused by high‐fat intake in rats. The sequencing results showed that a high‐fat diet significantly damaged the intestinal microbiota of rats, and MOC intake effectively reduced the negative effects of a high‐fat diet on the intestinal microbiota of rats. Blood lipids and intestinal microbiota are important components of human health. Studying the effect of MOC on blood lipids and intestinal microbiota is conducive to further application of MOC.

## AUTHOR CONTRIBUTIONS

**Yao Liu:** Methodology (equal); writing – original draft (equal). **Tengfei Xie:** Methodology (equal); writing – review and editing (equal). **Shijun Wu:** Methodology (equal); writing – review and editing (equal). **Guang Yang:** Writing – original draft (equal). **Jinyun Zhang:** Writing – original draft (equal). **Jie Song:** Writing – original draft (equal). **Guifang Yang:** Writing – original draft (equal).

## CONFLICT OF INTEREST STATEMENT

There is no conflict of interest.

## Data Availability

The data that support the findings of this study are openly available in figshare at https://doi.org/10.1002/fsn3.3490, reference number FSN3‐2023‐03‐0469.
